# EvolStruct-Phogly: incorporating structural properties and evolutionary information from profile bigrams for the phosphoglycerylation prediction

**DOI:** 10.1186/s12864-018-5383-5

**Published:** 2019-04-18

**Authors:** Abel Avitesh Chandra, Alok Sharma, Abdollah Dehzangi, Tatushiko Tsunoda

**Affiliations:** 1 School of Engineering & Physics, University of the South Pacific, Suva, Fiji; 2 Laboratory for Medical Science Mathematics, RIKEN Center for Integrative Medical Sciences, Yokohama, Japan; 3 Institute for Integrated and Intelligent Systems, Griffith University, Brisbane, Australia; 4 CREST, JST, Tokyo, Japan; 5 Department of Computer Science, Morgan State University, Baltimore, MD USA; 6 Department of Medical Science Mathematics, Medical Research Institute, Tokyo Medical and Dental University, Tokyo, Japan

**Keywords:** Post-translational modification, Protein sequence, Amino acids, Lysine, Phosphoglycerylation, Non-phosphoglycerylation, Predictor

## Abstract

**Background:**

Post-translational modification (PTM), which is a biological process, tends to modify proteome that leads to changes in normal cell biology and pathogenesis. In the recent times, there has been many reported PTMs. Out of the many modifications, phosphoglycerylation has become particularly the subject of interest. The experimental procedure for identification of phosphoglycerylated residues continues to be an expensive, inefficient and time-consuming effort, even with a large number of proteins that are sequenced in the post-genomic period. Computational methods are therefore being anticipated in order to effectively predict phosphoglycerylated lysines. Even though there are predictors available, the ability to detect phosphoglycerylated lysine residues still remains inadequate.

**Results:**

We have introduced a new predictor in this paper named EvolStruct-Phogly that uses structural and evolutionary information relating to amino acids to predict phosphoglycerylated lysine residues. Benchmarked data is employed containing experimentally identified phosphoglycerylated and non-phosphoglycerylated lysines. We have then extracted the three structural information which are accessible surface area of amino acids, backbone torsion angles, amino acid’s local structure conformations and profile bigrams of position-specific scoring matrices.

**Conclusion:**

EvolStruct-Phogly showed a noteworthy improvement in regards to the performance when compared with the previous predictors. The performance metrics obtained are as follows: sensitivity 0.7744, specificity 0.8533, precision 0.7368, accuracy 0.8275, and Mathews correlation coefficient of 0.6242. The software package and data of this work can be obtained from https://github.com/abelavit/EvolStruct-Phogly or www.alok-ai-lab.com

## Background

Post-translational modification (PTM) signifies the biological process responsible for the enzymatic change in proteins after its translation in the ribosome. There has been a stir of interest in these types of modifications across numerous organisms due to the progressive efforts of high-throughput proteomics in areas of site-specific PTM and protein altering enzymes [1]. Proteins are composed of 20 amino acids found in the genetic code. Lysine is one of the 20 amino acids which have been observed to be the most highly modified [2, 3]. According to the findings [4], lysine residues easily undergo covalent modifications and some of the modifications that have been detected are pupyl [5], propionyl [6], methyl [7], crotonyl [8], succinyl [9], glycosyl [10] and acetyl [11]. The modification of amino acids, as well as regulatory enzymes, have resulted in numerous human diseases including neurodegenerative disorders, rheumatic arthritis, coeliac disease, essential hypertension and high blood pressure, multiple sclerosis and coronary heart diseases.

This non-enzymatic phosphoglycerylation modification is found in human cells as well as in mouse liver [12]. Phosphoglycerylation is highly correlated to cardiovascular diseases due to it being linked to glycolytic process and glucose metabolism [13]. This reversible biochemical modification occurs as a result of the reaction between a primary glycolytic intermediate (1,3-BPG) and a lysine residue forming the 3-phosphoglyceryl-lysine (pgK) [14]. The 3-phosphoglyceryl-lysine hinders glycolytic enzymes and also accumulates on those that have their cells exposed to high glucose hence creating a potential feedback process causing the buildup and altering of glycolytic intermediates to different biosynthetic pathways. It is very crucial to understand the regularity roles and the selectivity mechanism of this PTM for the diagnosis and treatment of the affected individuals.

There has been an increasing interest in computational methods for predicting PTM sites in protein sequences [15–25]. It is because the experimental procedures for identifying PTM sites based in laboratories have demonstrated to be time-consuming, inefficient and a costly endeavor [26–28]. The computational technique of predicting phosphoglycerylated and non-phosphoglycerylated sites has proven itself to be an important tool for the identification process of such sites.

To address the computational technique of identifying the phosphoglycerylated lysine residues, some studies have been done previously. The Phogly-PseAAC is a KNN-based predictor which utilizes the pseudo amino acid properties with the center nearest neighbor algorithm [29]. CKSAAP_PhoglySite is another predictor which uses the method of Chou’s PseAAC and the k-spaced amino acid pair compositions (CKSAAP) [12]. The predictor employs penalty factor to treat the class imbalance and utilizes support vector machine to carry out prediction. It is intuitive to point out that the CKSAAP feature encoding scheme results in a very high dimensional feature vectors (2205 dimensional). Furthermore, it has been pointed out in a recent critical review [30] that this feature generation scheme does not perform well, hence it was not considered as an approach to take for the prediction of phosphoglycerylated lysine residues. The third method is called PhoglyPred [31]. This method uses sequence information obtained from the increment of k-mer diversity, the position-specific propensity of k-space dipeptide and finally selects physicochemical features of the modified k-space amino acid pair compositions. This method employs weight assignment on training data to solve the issue of class imbalance and then predicts the sites based on SVM classifier.

Despite the availability of a number of predictors, the capability in terms of performance is still very much of a concern. In this respect, we introduce an original predictor called EvolStruct-Phogly which employs a set of features comprising structural properties and evolutionary information for distinguishing phosphoglycerylated and non-phosphoglycerylated lysine residues. We have used 91 proteins containing phosphoglycerylated residues which have been experimentally identified and incorporated features such as the accessible surface area (ASA), probability of amino acid’s contribution to local structure conformations (coil, strand, helix), backbone torsion angles and profile bigram from the position-specific scoring matrix (PSSM) for all protein sequences. The residue window used in this work are different for the two property sets. The window size of ±3 proved to be significant for the structural properties while for the evolutionary information the window size of ±20. The stated window sizes provided the highest performance measures when segment sizes between 5 and 45 were assessed for each of the two characteristics (structural and evolutionary information). The feature vector, therefore, consisted of 3 upstream and 3 downstream and 20 upstream and 20 downstream amino acid residues for the two different characteristics corresponding to phosphoglycerylated and non-phosphoglycerylated sites. In the benchmark dataset, there existed a high class imbalance between non-phosphoglycerylated and phosphoglycerylated lysine residues hence we adopted the k-nearest neighbors strategy to carry out the cleaning action [26, 32, 33]. EvolStruct-Phogly showed a substantial improvement in the detection of phosphoglycerylated and non-phosphoglycerylated residues when compared with the existing predictors [12, 29] with sensitivity, specificity, precision, accuracy, and Mathews correlation coefficient equal to 0.7744, 0.8533, 0.7368, 0.8275 and 0.6242, respectively.

## Methods

A machine learning-based technique called EvolStruct-Phogly is proposed in this study for the prediction of phosphoglycerylated and non-phosphoglycerylated sites. This predictor considers a total of eight structural properties which are the ASA, the backbone torsion angles, and amino acid probability to local structure conformations (helix, strand, coil) [33, 34] and PSSM of proteins together with profile bigram [35] of amino acids for predicting phosphoglycerylated and non-phosphoglycerylated lysine residues. The following sections describes the benchmark dataset used in this work and acquisition of the characteristics of the segments consisting of the lysine residues.

### Benchmark dataset

For this work, the benchmark dataset was obtained from CPLM repository (http://cplm.biocuckoo.org). CPLM stands for Compendium of Protein Lysine Modifications and holds a number of other protein lysine modifications which have been experimentally determined. In order to use the dataset, we removed those protein sequences which had ≥40% sequential similarities. The consequent number of proteins attained was 91 and each of the sequences contained one or more lysine residues. A total of 3360 lysine residues were found in these protein sequences and out of which 3249 lysines were non-phosphoglycerylated. The following sections describe the computation of the two characteristics of the protein sequences used in this work.

### The structural and evolutionary features

#### Structural features

The structural features attained in this work corresponded to eight properties which are the secondary structure, the ASA and the backbone torsion angles. SPIDER2 toolbox [36] was used to achieve the mentioned properties. The SPIDER2 toolbox is compatible for accomplishing good result in predicting the secondary structure [37, 38], the ASA [39, 40] and the backbone torsion angles [39, 41] in protein sequence. The toolbox can successfully extract structural properties for sequence-based binding sites of proteins [42, 43]. Structural properties are further elaborated in the subsections below. For simplicity, we call the below feature matrix as *SPpre.*

##### Accessible surface area

ASA is the approximation of an amino acid’s accessible area to a solvent [44, 45]. It reveals essential information about the protein structure of individual amino acids. The resulting ASA value of individual amino acids was obtained by executing SPIDER2 on every protein sequence. It can be pointed out here that SPIDER2 predicts upon the primary sequence hence the prediction is entirely based on the sequence information.

##### Secondary structure

The 3D structure of proteins is defined by the secondary structure. Predicted secondary structure gives a distinct outcome contributing to either coil, strand or helix, which are the protein local structures. SPIDER2 was used again to evaluate the occurrence of amino acid conformations to the local structures; coil (*pc*), strand (*pe*) and helix (*ph*). The result of SPIDER2 is an L × 3 matrix, where L denotes the protein length while the columns denote the conformation probability to the three secondary structures.

##### Local backbone angles

Local backbone angles, also known as torsion angles, relates the neighboring amino acids. The torsion angles ϕ, and ψ, corresponding to a local amino acid are a measure representing its interaction along the protein backbone [46, 47]. For each amino acid, the angle ϕ_i_ specifies the dihedral angle for the N_i_ - Cα_i_ bond while ψ_i_ is the angle spun about Cα_i_ - C_i_ bond. In the recent works, the inclusion of two new angles has been focused which are based upon dihedral angles θ, the angle between three Cα atoms Cα_i-1_ - Cα_i_ - Cα_i + 1_ and τ, the angle rotated about Cα_i_ - Cα_i + 1_ bond, have been considered [39]. Four different numerical vectors ϕ, ψ, θ, and τ were achieved corresponding to each amino acid after running the SPIDER2 toolbox. Torsion angles complement ASA and secondary structure through the provision of important continuous information of amino acid’s local structure [41].

#### Evolutionary feature

The underlying insights of how the proteins evolved based on its structural, functional and sequential similarities with others [48] are captured by evolutionary information. For each amino acid in the protein, PSSM provides the probability of substitution with the 20 amino acids found in the genetic code. PSI-BLAST is a toolbox [49] that aligns a given protein sequence to similar sequences located in the protein data bank [50] was used to obtain the PSSM. The PSSM of proteins in our benchmark dataset was obtained by running the PSI-BLAST tool. Two matrices are outputted by PSI-BLAST of the dimension L × 20 where L corresponds to the protein length and columns to the 20 amino acids found in the genetic code. One matrix represents the log odds and the second matrix the amino acid linear probabilities. The linear probabilities were employed for the purpose of this work. PSSM was produced on non-redundant proteins by PSI-BLAST in the protein data bank for three iterations using E value (cutoff value) of 0.001.

### Formulation of the amino acid characteristics

**Fig. 1:**
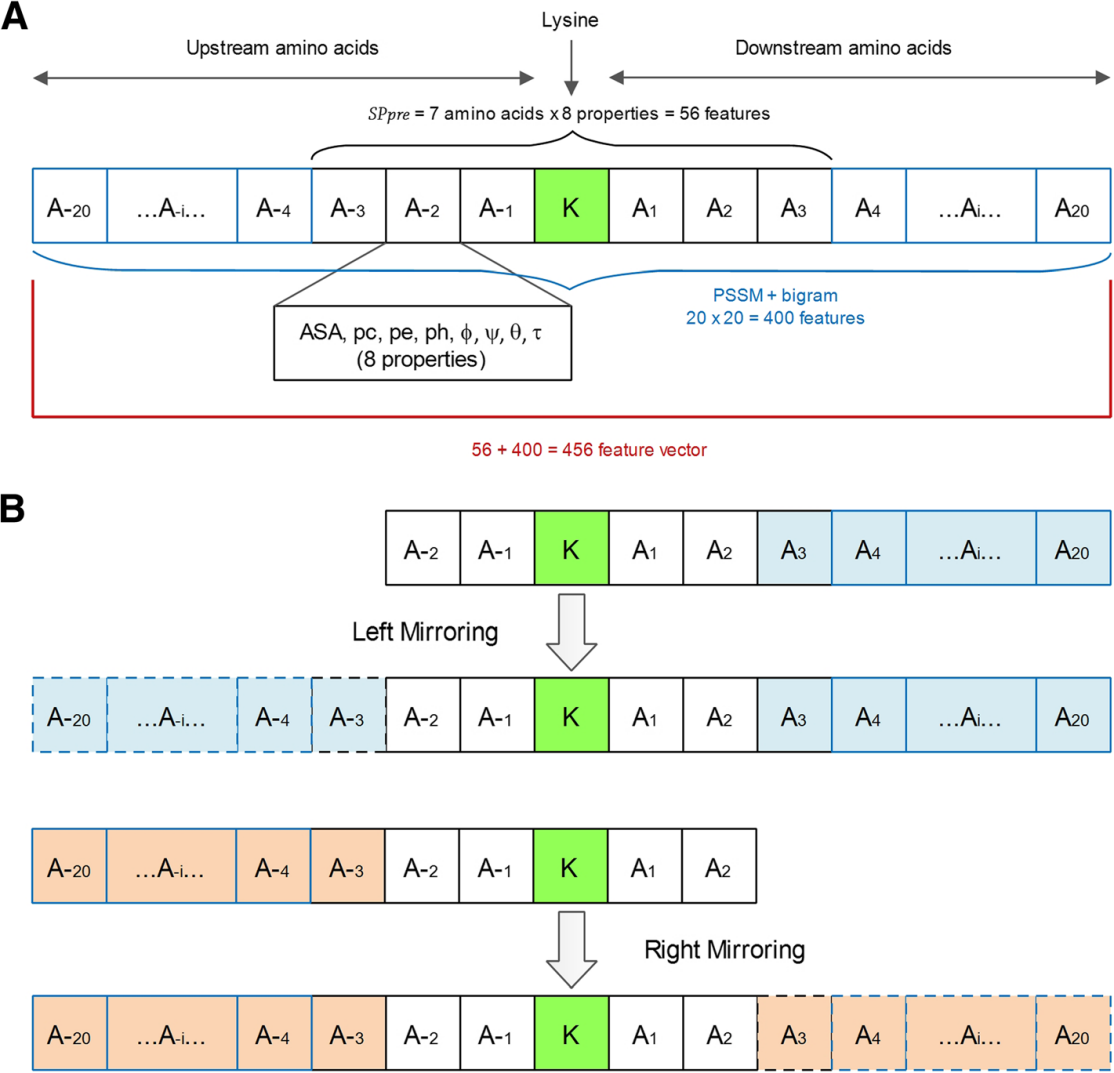
Schematic representation of the arrangement of lysine residue amino acid neighbors. **a** Lysine residue possessing sufficient neighboring amino acids. **b** Exemplar lysine residue having insufficient amino acids. The application of left and right mirroring for insufficient upstream and downstream amino acids respectively

In this section, we will look into the formulation of the structural properties (ASA, *pc*, *pe*, *ph*, ϕ, ψ, θ, τ) and the evolutionary information for each lysine residues. We have utilized 3 upstream and 3 downstream amino acids for structural features and 20 upstream and 20 downstream amino acids for evolutionary feature surrounding the lysine residue *K* as shown in Fig. 1a. In the circumstances where the lysine residues had missing amino acids on the upstream or downstream, the technique of mirror effect [33] was employed to fill in the missing amino acids as depicted in Fig. 1b.

A segment P comprising 20 upstream and 20 downstream amino acids including the lysine residue *K* which falls in the middle can be written as:

1 $$ P=\left\{{A}_{-20},\dots, {A}_{-2},{A}_{-1},K,{A}_1,{A}_2,\dots, {A}_{20}\right\} $$

The downstream amino acids are referred by *A*_*n*_ where 1 ≤ *n* ≤ 20 and upstream amino acids by *A*_*-n*_ where 1 ≤ *n* ≤ 20. It can be realized from eq. (1) that a segment is made up of 41 amino acids (20 upstream amino acids, 20 downstream amino acids and the lysine *K*). The segment P represents each lysine and a label of 1 indicates phosphoglycerylation site and a 0 indicates the non-phosphoglycerylated site. These labels are experimentally confirmed valuations.

Furthermore, after the acquisition of the sub-matrices PSSM and *SPpre*, PSSM was changed into frequency vector of bigrams (PSSM + bigram) and after which the computed features were used to describe each lysine site. Resulting dimensions of the matrices were 7 × 8 (*SPpre*) and 20 × 20 (PSSM + bigram). The segment P corresponding to each lysine residue was therefore composed of a 456 dimensional vector. All in all, the 456 dimensional feature vector captured the structural properties and evolutionary information for the segment P representing each lysine residue.

### Profile bigrams

The technique of profile bigrams has presented promising outcome when dealing with discriminatory information [35, 51–53]. The matrix M represents PSSM for each protein sequences. Every element in the matrix M denoted by m_ij_ is the transitional probability of amino acid j at the i-th position of the protein sequence. Segment P was represented by a feature matrix of size 41 × 20 where 20 indicates amino acids of the genetic code from which PSSM has calculated the substitution probabilities of each amino acid. Profile bigram [35] of the matrix M is calculated by

2 $$ {B}_{p,q}={\sum}_{k=1}^{40}{m}_{k,p}{m}_{k+1,q}\kern0.50em \mathrm{where}\ 1\le p\le 20\ \mathrm{and}\ 1\le q\le 20 $$

The matrix *B* comprises of elements *B*_*p*, *q*_ (for *p* = 1, 2, 3, …, 20 and *q* = 1, 2, 3, …, 20) representing PSSM + bigram is a 20 × 20 matrix. The matrix *B* is then transformed into 400 transitional probabilities as shown by Eq. (3). The feature vector contains 400 transitional probabilities corresponding to the evolutionary information of each lysine residue.

3 $$ F=\left[{B}_{1,1},{B}_{1,2},\dots, {B}_{1,20},{B}_{2,1},{B}_{2,2},\dots, {B}_{2,20},{B}_{20,1},{B}_{20,2},\dots, {B}_{20,20}\right] $$

### Support vector machine

Support vector machine is a collection of learning algorithms categorized under the supervised learning model in the area of machine learning. The model is useful for analyzing data for classification and regression applications. Each training data point resembles a point in the n-dimensional space where n is the number of features of the sample. The way the SVM algorithm works is by finding a hyper-plane which best separates the two different classes. The classes are not always linearly separable so the non-linear kernels are employed to deal with such cases. The kernels are used to map nonlinear input space to a higher dimensional feature space in which the classes can be linearly separated. For this work, LibSVM package was utilized on the Matlab platform. Furthermore, the SVM type used was C-SVC and kernel employed was polynomial with the cost value of 1 and gamma value of 1.

## Results and discussion

Getting the performance assessment of any predictor intended for predicting phosphoglycerylation sites is a very important component. For the purpose of this work, we have used five different statistical metrics to evaluate the performance of EvolStruct-Phogly. The metrics are sensitivity, specificity, precision, accuracy and Mathews correlation coefficient [12, 26, 29, 33, 54–58]. The following sections discuss the evaluation metrics used, the validation scheme, the procedure for treating class imbalance and finally the comparison of EvolStruct-Phogly with existing methods.

### Evaluation metrics

The first metric sensitivity, measures the ability of the predictor to correctly classify phosphoglycerylation lysine residues. The range of values of the metric is from 0 to 1 where a 1 indicates a very effective predictor and a value of 0 shows that the predictor is incompetent. In other words, a higher value of sensitivity metric indicates the better the predictor is at distinguishing phosphoglycerylation sites.

Specificity is the second metric which is the measure of a predictor to classify correctly the non-phosphoglycerylation sites. This metric also ranges from 0 to 1 where a higher value signifies the better the predictor is at distinguishing non-phosphoglycerylation sites.

The third metric is precision and it indicates the portion of the entire predicted phosphoglycerylated residues by the classifier to be correctly classified. The metric provides a measure of the predictor’s ability not to label a site as phosphoglycerylated if the site is actually non-phosphoglycerylated. The metric values range from 0 to 1 where 1 is the most desired score while 0 is not.

The fourth metric is accuracy and it captures the ability of a predictor to distinguish phosphoglycerylated sites from non-phosphoglycerylated ones. It is calculated by dividing the sum of predicted phosphoglycerylated and non- phosphoglycerylated sites which reflect the true labels with the total number of sites predicted. The metric values also range from 0 to 1 where 1 is the most desired score while 0 is not.

The final metric is known as Mathews correlation coefficient [59] and is used for measuring the quality of a two-class classifier. It is considered to be a balanced measure since it can be utilized even when the two classes are of very different sizes. This metric ranges between − 1 and 1. A score of 1 indicates a very competent predictor, 0 as an average predictor while a − 1 as an impractical predictor.

The five evaluation metrics can be summarized as

4 $$ Sensitivity=\frac{TP}{TP+ FN} $$

5 $$ Specificity=\frac{TN}{TN+ FP} $$

6 $$ Precision=\frac{TP}{TP+ FP} $$

7 $$ Accuracy=\frac{TN+ TP}{FN+ FP+ TN+ TP} $$

8 $$ Mathews\ correlation\ coefficient=\frac{\left( TN\times TP\right)-\left( FN\times FP\right)}{\sqrt{\left( TP+ FP\right)\left( TP+ FN\right)\left( TN+ FP\right)\left( TN+ FN\right)\ }} $$

where *TP* stands for true positives corresponding to the phosphoglycerylated lysines correctly predicted. *TN* stands for true negative samples which corresponds to the number of non-phosphoglycerylated residues correctly predicted. *FN* denotes false negatives representing the samples which were phosphoglycerylated but were predicted as non-phosphoglycerylated sites. The *FP* is a number of false positive samples which is the number of non-phosphoglycerylated sites incorrectly classified by the predictor.

It is preferred that the best predictor must achieve highest scores in all the mentioned evaluation metrics. Nevertheless, the performance of the predictor on the sensitivity measure should be higher than the existing methods.

### Validation scheme

The evaluation metrics described in the previous section were obtained through cross-validation method so that the performance of the predictor can be deduced. The three commonly used cross-validation methods to determine predictor’s effectiveness in statistical predictions are independent dataset test, n-fold cross-validation test, and jackknife test [60, 61]. Even though the jackknife is the least arbitrary of the three methods yielding the distinct result for a given dataset [62], the 10-fold cross-validation method was adopted in this work to reduce the computational time. The steps in which the 10-fold cross-validation method was performed is highlighted below:

1. Split the dataset into the folds of 10 where each fold is of equal size
2. Carry out training on the 9 folds and test on the remaining fold
3. Fine-tune the parameters of the predictor on the training sets
4. Calculate the five evaluation metrics on the test fold
5. Reiterate the steps 2 to 4 for nine more epochs and calculate evaluation metric averages.

The result for the 10-fold cross-validation carried out in this work is presented under the section where the comparison with existing methods is shown.

### Data imbalance treatment

In the obtained benchmark dataset, it was discovered that the number of phosphoglycerylated lysine residues was much less compared to the number of non-phosphoglycerylated lysine residues. The number of positive samples (phosphoglycerylated sites) was 111 while the number of negative samples (non-phosphoglycerylated sites) was 3249. As a result, the ratio obtained between positive and negative sets was 1:29 which could strongly bias the classification process. For this reason, dealing with class imbalance is a very crucial action in classification problems. To carry out the imbalance treatment, we utilized the commonly used scheme called the k-nearest neighbor strategy [26, 28, 32, 55, 63] where we removed a negative instance when one of its k neighbors was a positive instance. We started out the process by finding the initial value of k by dividing the number of samples in the negative set with the number of samples in the positive set. The resulting value of k obtained was 29. We then calculated the Euclidean distance of all the samples from every negative sample and removed the negative sample when one of its neighbors was positive. With the k value of 29, it was found that the class imbalanced remained. The threshold was therefore increased further until the negative set was about twice the size of the positive set. A k value of 79 resulted in 226 samples in the negative set and 111 samples in the positive set. It is to be noted that the number of samples in the positive set was not modified in the treatment process. The resulting samples were then employed to deduce the performance of the new predictor based on the 10-fold cross-validation method.

### Comparison of EvolStruct-Phogly with the existing methods

The two recently developed techniques for predicting phosphoglycerylated sites are the Phogly-PseAAC [29] predictor and the CKSAAP_PhoglySite method [12]. We uploaded our benchmark dataset in FASTA format to the webserver of the Phogly-PseAAC predictor to obtain their classification results. It is worthy to point out that the webserver could have been trained using some of the protein sequences which are being used for the performance evaluation. For the second method, the Matlab software was provided for predicting the phosphoglycerylated sites in protein sequences. In order to carry out the comparison with the CKSAAP_PhoglySite predictor, we built the feature extraction of the lysine residues using their technique and performed the same 10-fold cross-validation on the classifier similar to ours. For both of these methods, evaluation was carried out using the same validation set which was put aside when 10-fold cross-validation was performed on our predictor EvolStruct-Phogly. Furthermore, we computed the area under the curve (AUC) for 10-fold cross-validation of our predictor and the method of CKSAAP_PhoglySite. AUC could not be calculated for the Phogly-PseAAC predictor since the training samples used in their method was not clear.

Table 1 shows the comparison of EvolStruct-Phogly, Phogly-PseAAC predictor [29] and the CKSAAP_PhoglySite method [12]. It can be seen that EvolStruct-Phogly outperforms the other two methods in the metrics which are sensitivity, precision, accuracy, and MCC (Mathews correlation coefficient). The four metrics improved significantly by 11.2, 29.3, 15.3 and 51.6%, respectively, with respect to the highest value of each metric. This goes on to say that there is a considerable improvement over the previous methods. It can be noted that even though the specificity of the CKSAAP_PhoglySite method [12] remained high (0.9327), its sensitivity was quite low (0.1724), leaving almost 83% of phosphoglycerylation residues undetected. Moreover, the AUC of EvolStruct-Phogly and CKSAAP_PhoglySite method [12] were computed to be 0.8144 and 0.5524, respectively. Predictor having a higher value of AUC is always favorable.

**Table 1:** Comparison of the two benchmark prediction methods with EvolStruct-Phogly predictor using 10-fold cross-validation procedure

Method	Sensitivity	Specificity	Precision	Accuracy	MCC
CKSAAP_PhoglySite method [12]	0.1724	**0.9327**	0.5500	0.6822	0.1645
Phogly-PseAAC [29]	0.6962	0.7299	0.5697	0.7178	0.4117
EvolStruct-Phogly	**0.7744**	0.8533	**0.7368**	**0.8275**	**0.6242**

Metric highlighted in bold indicate the highest value

It can be seen from the results that EvolStruct-Phogly has delivered a very promising performance. The promising performance can be credited to the usage of important structural properties and evolutionary information concealed in the protein sequences. The combination of structural properties such as the ASA of amino acid, local structure conformations, backbone torsion angles and the evolutionary information captured by PSSM of each amino acid which was translated to bigram occurrences appear to be vibrant characteristics in terms of detecting the phosphoglycerylated residues. The use of structural properties and evolutionary information has propitiated other areas of research like subcellular localization of proteins [64], succinylation prediction [33], MoRF detection [65, 66], and protein fold recognition [67].

Furthermore, we calculated the absolute of Pearson correlation between the structural properties, coil (*pc*) and strand (*pe*), for the positive samples, negative samples, and combined positive and negative samples. The correlation coefficient obtained were 0.0979, 0.0819 and 0.0234, respectively. It can be seen that there is a higher correlation in the positive and negative sets for the structural properties coil and strand when compared to that of the combined set.

A user-friendly web-server which is publically accessible, as indicated in [68] and also in a series of latest publications (see, e.g., [65, 69–74]), represents the steps ahead for developing prediction methods and computational tools which are more practical and useful. We therefore, in our future works, shall make efforts to provide a web-server for the prediction method presented in this paper.

## Conclusion

To sum up, a new predictor called EvolStruct-Phogly is presented in this paper which employs a combination of structural properties and evolutionary information for predicting phosphoglycerylated lysine residues. The profile bigram was computed for the evolutionary information of proteins and was integrated with structural properties to form a single vector to carry out the classification. There was a high class imbalance in the benchmark dataset which was treated using the k-nearest neighbors technique and was then supplied to the SVM classifier for phosphoglycerylation site prediction. With our method, the sensitivity, precision, accuracy, and MCC significantly improved when compared to the previous predictors.

## Abbreviations

ASA: Accessible surface area
AUC: Area under the curve
CPLM: Compendium of Protein Lysine Modifications
MCC: Mathews correlation coefficient
PSSM: Position-specific scoring matrix
PTM: Post-translational modification
SVM: Support vector machine
